# The Chemist Who Stayed in Gaza

**DOI:** 10.1021/acscentsci.4c01418

**Published:** 2024-09-12

**Authors:** Laurel Oldach

In May, while most of the civilian population of the Gaza Strip was preparing to flee the southern city of Rafah, Rami Morjan was planning to go there.

**Figure:**
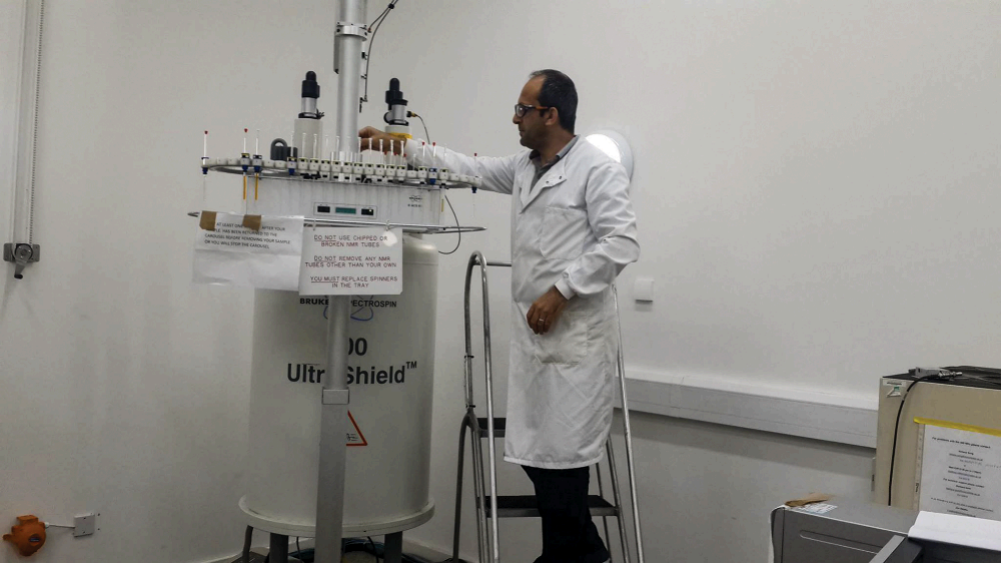
Rami Morjan uses a nuclear magnetic resonance spectrometer during a visit to a collaborator’s lab outside the Gaza Strip in an undated photo taken before the war. Because analytical instruments could not be imported into Gaza, Morjan relied on collaborations to complete and publish his research. Credit: Courtesy of Rami Morjan.

The city, which for a time had been a humanitarian safe zone, was refuge for an estimated 1.2 million people. But in late April, the Israeli army announced plans to enter Rafah, a move it said was critical to destroying Hamas. It was a dangerous place for civilians. But Morjan had family there who could not leave: his sister and her family, including a niece and her newborn baby.

## Hoping to survive

Morjan, an organic chemist at the Islamic University of Gaza, is used to facing overwhelming odds. He built a chemistry research program despite lacking tools for chemical analysis and then rebuilt it after his laboratory was bombed a decade ago. In May, even after having endured 7 months of war, he came across in text conversations with *C&EN* as lighthearted, peppering chats with the “face with tears of joy” emoji.

He aims to rebuild the academic community—especially the chemistry community—in Gaza after the war. But first, he must survive it.

In its attack on Israel in October 2023, Hamas killed an estimated 1,200 people and took about 250 people hostage. In answer, Israel launched a military response that has killed more than 37,000 Palestinians, according to Gaza’s Ministry of Health. A further 10,000 are missing, according to the Palestinian Civil Defense, and the war has displaced 75% of the Gaza Strip’s over 2 million residents, per the United Nations. The BBC reported in January that at least half of Gaza’s buildings had been damaged or destroyed by that time.

Research and teaching have come to a halt, along with most other functions of civil society. Cell phone service is unreliable, as are supplies of food, water, and power. Colleagues who live in the West Bank say that when someone in Gaza drops out of touch, it is impossible to determine whether they are alive or have been killed. Some chemists whom *C&EN* contacted were unwilling to speak on the record because of concern about their safety.

**Figure:**
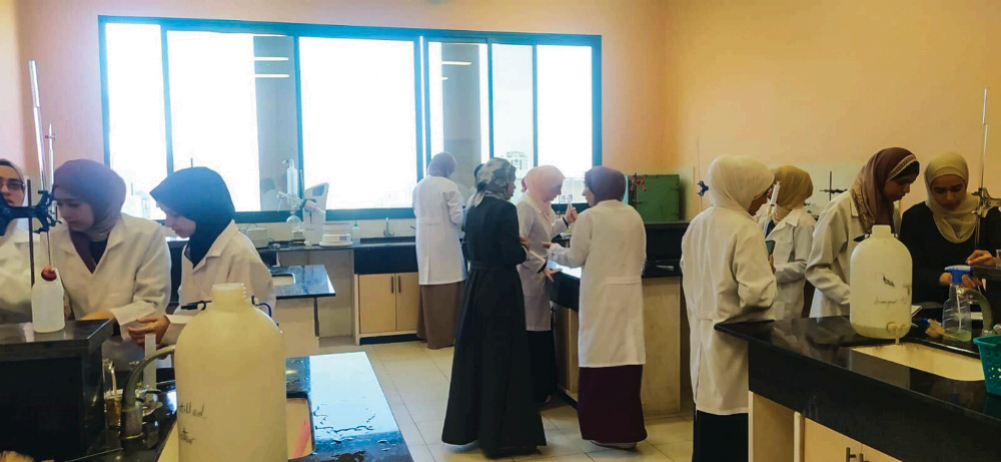
Students learn chemistry in Rami Morjan’s lab at the Islamic University of Gaza in an undated photo from before the war. Credit: Courtesy of Rami Morjan.

*C&EN* interviewed Morjan over a period of 4 weeks, using typed messages on the messaging platform WhatsApp. All quotations attributed to Morjan are taken from those messages. At that time, Morjan was in the coastal town of Deir al-Balah. He had been there since October, sharing a single room with his wife and four children. He had regained reliable Internet access only recently, he wrote on May 2. “To be honest as I (we) lost everything since Oct 7 and the genocide still going on we all were engaged in one thing which is (will we survive)?”

International legal scholars have argued over whether Israel’s actions constitute genocide, including in a pending case before the International Court of Justice. A report from the University Network for Human Rights concludes that Israel’s actions since October violate the Convention on the Prevention and Punishment of the Crime of Genocide of 1948. Israel has strongly rejected these accusations.

While those debates have unfolded, Morjan has focused on more immediate concerns. “This is our daily life just to secure the food and water,” Morjan wrote. Because nowhere feels safe, he added, “we spend most of our time at home and hoping to survive.”

**Figure:**
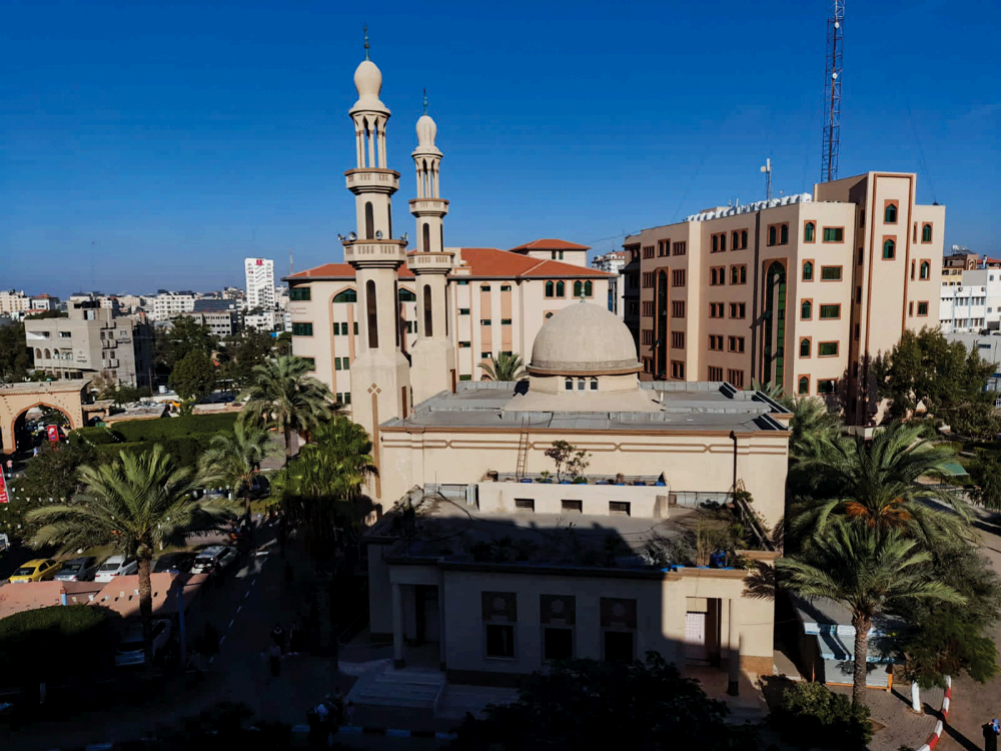
The campus of the Islamic University of Gaza in an undated photo Rami Morjan took before the war. Credit: Courtesy of Rami Morjan.

## Before the war

Morjan was accustomed to facing challenges performing chemical research in Gaza.

“He did an amazing job being able to run some chemical research,” says John Gardiner, Morjan’s former PhD adviser and longtime collaborator. Morjan arrived in his lab at the University of Manchester in 2000 with little experience but determined to learn, and he was hardworking and productive, Gardiner says.

Morjan faced more barriers to research after he graduated and returned to the Gaza Strip. He became a professor at the Islamic University of Gaza (IUG) in 2006.

“Education has always been central to life in Gaza,” says Sultan Barakat, a public policy professor at Hamad Bin Khalifa University in Qatar who specializes in conflict and reconstruction and has studied the Gaza Strip for decades. He adds that Palestinians believe that education is one of the few assets that cannot be destroyed by bombing.

The IUG had a prominent place within that education system. It was Gaza’s largest and—depending on how you count a nearby former teacher’s college—oldest university. It also held a complex place in Gaza’s politics.

The year 2006, when Morjan became a professor at the university, was the same year Hamas won a majority in parliamentary elections. After a violent struggle with another political party, the group seized control of the territory and has ruled it since 2007.

“Today it is common to refer to IUG as Hamas-affiliated,” Erik Skare, a postdoctoral scholar at the University of Oslo who studies political movements in Palestinian history, writes in an email to *C&EN*. “Yet, and this is important, not everyone involved as student or faculty at the IUG has an affiliation with Hamas. IUG represents the diversity of Palestinian society just as the University of Toronto represents the diversity of that city.”

Whether students and faculty supported Hamas or not, being part of the university community put them in the crosshairs. Hamas’s hostilities with Israel erupted into armed conflict several times between 2006 and the start of the current war in October 2023. Israel bombed the campus in 2008, 2014, and 2021. After the 2014 bombing destroyed the science building, Gardiner says, Morjan helped raise money to rebuild. “They’d really only in the last couple of years got back to where they had been,” Gardiner says.

Still, some types of research were out of reach. Power was generally available only 8–12 h a day; materials and equipment were in short supply. Blockades meant that people in Gaza could not legally import chemicals or—according to a nongovernmental organization’s unofficial translation from Hebrew—“equipment and tools of physical and chemical analysis.” So scientists had to adapt protocols for the available materials and equipment.

Morjan focused on synthesizing heterocycles, with an interest in their biological properties. When his students completed a synthesis, he said, they could analyze the product’s melting point or conduct thin-layer chromatography. For more sophisticated analysis, they had to send a sample abroad.

International collaborations enabled Morjan and his students to coauthor 17 research papers in the past decade. But, Gardiner says, “it was difficult and still well below the freedom to operate on a time scale that would be true outside Gaza.”

Amani Ahmed, an administrator at the IUG, says that in Gaza, “every person or every institution needed to double or triple the efforts that any regular person is doing worldwide.”

Ahmed’s office for international affairs connected researchers like Morjan to international academic and nongovernmental organization partners. Over the years, she and her team set up 300 projects, she says, including student and faculty exchange programs, joint research activities, and curriculum redesign.

In 2018, Morjan became chair of the chemistry department and tackled a series of projects. He organized a chemistry conference for the Palestine Academy for Science and Technology that colleagues attended remotely because travel between Gaza and the West Bank was forbidden.

He also conducted outreach to high school students interested in science. To raise enrollment in chemistry, he started a detergent factory, using the proceeds from soap sales to offer scholarships. One of his students, Jannat Azzara, who had worked with Morjan as an undergraduate and master’s student, volunteered to help. Over time, they expanded the project, offering free hand sanitizer during the COVID-19 pandemic and science kits for local schools.

In June 2023, the IUG recognized Azzara with an award for best master’s graduate in chemistry. In a post on Facebook that month, she thanks Morjan for his support and encouragement of students. But by the end of the year, she was dead. Morjan wrote, “My university was demolished and my right hand agent has been killed and every thing gone.”

## Toll on education

Azzara died in the bombardment of the city of Gaza in October, Morjan says. (*C&EN* found her married name, Jannat Harbawi, on a list of 30 people who died in an air strike at her husband’s family home on October 13.)

An air strike destroyed Morjan’s university that same month. A spokesperson for the Israel Defense Forces tells *C&EN* in an emailed statement, “Both the university building and its surroundings were used by Hamas for various military activities, above and below ground, this includes a development and production of weapons and the training of the intelligence personnel in the military branch of Hamas.”

**Figure:**
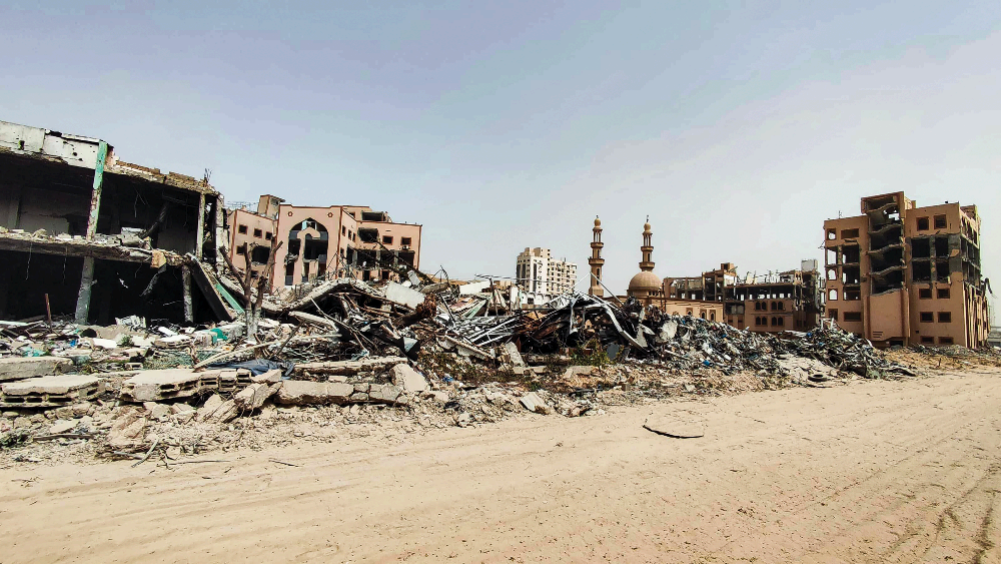
A view of the campus of the Islamic University of Gaza on April 18, after it was destroyed in Israeli air strikes in October. Credit: Khaled Daoud apaimages.

Sources in the West Bank and Gaza were reluctant to speak on the record about Hamas. Skare, the political movement researcher in Norway, writes that although Israel has repeatedly claimed that Hamas used the IUG campus, “these allegations have never been substantiated and independent investigations need to be carried out.” Moreover, he adds, “it is not just IUG that has been targeted.” Israel’s military has damaged all of Gaza’s universities; most are completely destroyed. A panel of experts from the United Nations raised concern in April that Israel could be deliberately targeting institutions of higher education. The Israeli army says in a statement to *C&EN* in response, “Hamas makes systematic military use of public buildings that are supposed to be used for civilian purposes only, including educational institutions and universities.”

## Better than nothing

Morjan, however, was determined to keep teaching, even without a lecture hall or labs. When he regained access to the Internet in early May, he began live streaming lectures in chemistry, joining an effort by universities in the West Bank to provide an interim education to students from Gaza. Charging his phone with a solar energy unit, he would stand in the middle of the street to get a strong enough signal. For his students, he said, “it’s [a] little thing that might be better [than] nothing.”

**Figure:**
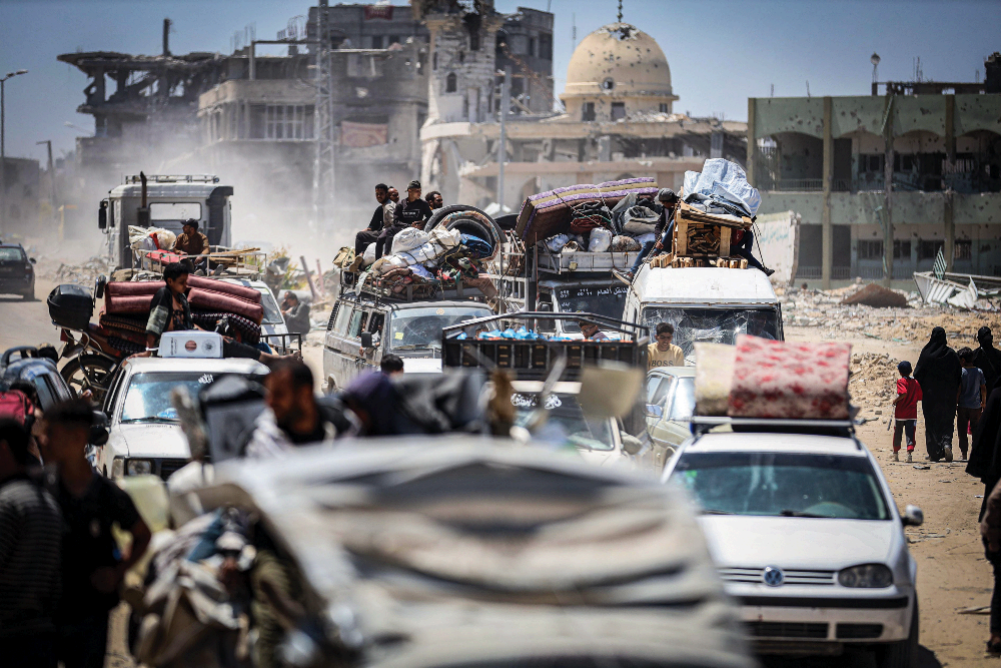
Palestinians evacuating from Rafah, the Gaza Strip, return to the battered Gazan city of Khan Yunis on May 7. Credit: Saher Alghorra/ZUMA Press Wire.

In early May, Israeli troops entered Rafah, where Morjan’s sister and her family were. Hundreds of thousands of internally displaced people fled from what had been the Gaza Strip’s safest city to tent camps in areas with even less infrastructure. Israel closed the border crossing between Egypt and Gaza, cutting off both emigration and a major route for incoming humanitarian supplies.

Many students stopped attending Morjan’s virtual lectures. He thought the vast majority had been in Rafah and were preoccupied with surviving, not attending chemistry class. With his usual light touch, Morjan wrote, “I am not convinced with e learning!!! I am counting days for the war to stop so we can physically teach.”

Meanwhile, while others fled the area, Morjan made his trip from Deir al-Balah to Rafah, hoping to give his sister’s family what help he could. At least the baby had arrived safely, he wrote. But the situation was bad. “I couldn’t find healthy food for her no meat, no chicken, the tray of eggs cost about 50$,” he wrote. “It’s really hard time.”

The situation was calmer when Morjan returned to Deir al-Balah, where his wife and children had stayed. “In general is quiet if we ignored few attacks some where,” he said on May 14. But it was badly overcrowded, without infrastructure to support the displaced population.

At the end of May, a few days after an air strike on a tent camp in Rafah ignited a fire that killed at least 45 people, Morjan left Deir al-Balah again and went back to help his sister and her family evacuate. When they returned, because there was no space in the house Morjan and his family were already crowded into, his sister and her family went to a tent in an open area.

**Figure:**
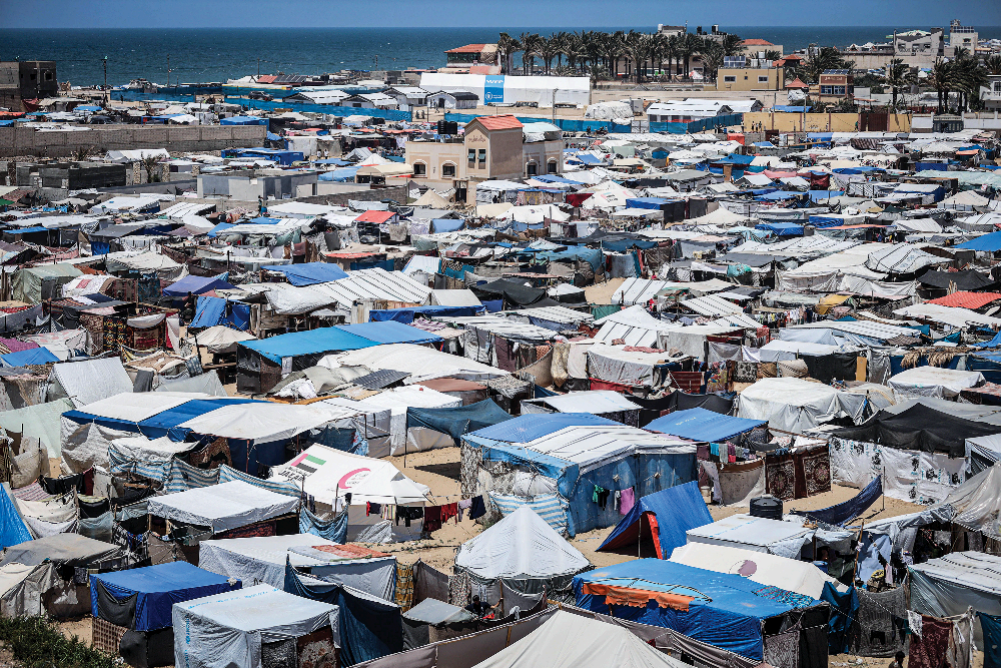
In May, hundreds of thousands of internally displaced Palestinians fled Rafah, the Gaza Strip, after Israel announced it would begin a military operation there. Many went to the Gazan city of Deir al-Balah (shown in this May 12 photo), where Rami Morjan and his family had been staying. Credit: Saher Alghorra/ZUMA Press Wire.

On May 14, Morjan reflected on his choice not to emigrate earlier in the war, when the opportunity was there. “You know, all my brothers and two of my sisters and my mother have left Gaza and I decided not to leave on the hope to re building if I survive,” he wrote. “I had the chance to leave. But I rejected it.”

Whenever peace might be established, Morjan wrote, he hopes to launch a massive, nonpolitical crowdfunding project called Chemists for Gaza to rebuild razed laboratories. He hopes to rebuild his chemistry department, again, and to get back to teaching students in person. He said of these students, “These educated individuals will help spread principles of love, peace, progress, and prosperity, fostering a more humane world.”

**Figure:**
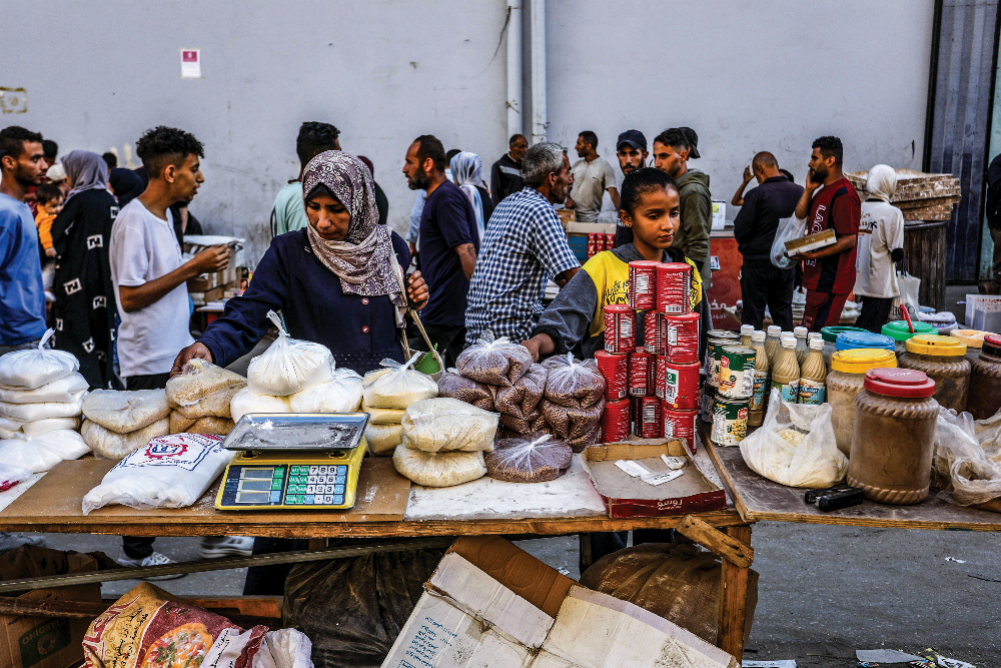
In mid-May, food was still available, but expensive, at open-air markets like this one in Deir al-Balah, the Gaza Strip. Credit: Abed Rahim Khatib/dpa/Alamy Live News.

Though so much that he worked to build had been destroyed, Morjan wrote in mid-May, “[I] still hope the crazy war will stop and I will be able to start again.”

But by May 30, he sounded much less hopeful. Thinking back on his lab’s research before the war began brought him to tears, he said. “I am sad because it will not come back. It seems Gaza is no more a place to live.”

*With additional reporting by Rasha Faek, special to C&EN. Laurel Oldach is an associate editor at*Chemical & Engineering News, *the independent news outlet of the American Chemical Society. A version of this story appeared in C&EN on June 17, 2024. Since then, the war in Gaza has continued, with the death toll reported by Gaza’s Ministry of Health rising past 41,000 people. Other figures in this story may be out of date as well*.

*Read more C&EN articles about the war in Gaza and its effects on chemistry:*

Gaza’s universities are gone. What’s next for science education?

Editorial: Antisemitism impoverishes science education and research

